# Design and Analysis of a Dynamic Mobility Management Scheme for Wireless Mesh Network

**DOI:** 10.1155/2013/656259

**Published:** 2013-11-07

**Authors:** Abhishek Majumder, Sudipta Roy

**Affiliations:** ^1^Department of Computer Science & Engineering, Tripura University, Suryamaninagar, 799022 Tripura West, India; ^2^Department of Information Technology, Assam University, Silchar, 788011 Assam, India

## Abstract

Seamless mobility management of the mesh clients (MCs) in wireless mesh network (WMN) has drawn a lot of attention from the research community. A number of mobility management schemes such as mesh network with mobility management (MEMO), mesh mobility management (M^3^), and wireless mesh mobility management (WMM) have been proposed. The common problem with these schemes is that they impose uniform criteria on all the MCs for sending route update message irrespective of their distinct characteristics. This paper proposes a session-to-mobility ratio (SMR) based dynamic mobility management scheme for handling both internet and intranet traffic. To reduce the total communication cost, this scheme considers each MC's session and mobility characteristics by dynamically determining optimal threshold SMR value for each MC. A numerical analysis of the proposed scheme has been carried out. Comparison with other schemes shows that the proposed scheme outperforms MEMO, M^3^, and WMM with respect to total cost.

## 1. Introduction

Now-a-days wireless mesh network (WMN) [1, 2] has emerged as one of the promising technologies for providing network connectivity to increasing number of mobile users. Moreover, due to advantage of cost effectiveness, robustness, and easy and fast deploy ability; it has become an attractive technology for future network implementation. 

WMN consists of three types of nodes: mesh client (MC), mesh router (MR), and gateway (GW). MCs are the mobile users of the WMN. MRs are the wireless routers used for routing of packets from one mesh node to another. An MR having a wired interface to the Internet is called GW. There are two kinds of traffic that flows in the WMN: Internet and Intranet. The Internet packets pass through the GW. The GW receives the downstream Internet packets and sends those to the destination MCs through WMN. In case of upstream Internet traffic, packets are sent from the MC to the GW. On the other hand, Intranet communication takes place between two MCs of same WMN. 

One of the major problems in WMN is provisioning of seamless network connectivity for the MCs as it moves from one MR to another. For solving this problem, several mobility management techniques such as MEsh networks with MObility management (MEMO) [3], Mesh Mobility Management (M^3^) [4], and Wireless mesh Mobility Management (WMM) [5] have been proposed. MEMO [3] restricts transmission of control message in the WMN to reduce control overhead of the network. But, if the mobility of the MC is high, more numbers of control packets are transmitted by the MCs. So, mobility is an important characteristic of MC. To reduce the control overhead the concept of forward chain has been introduced in M^3^. Packets are forwarded through the forward chain. But, in case of a network where session arrival and departure rate to and from the MC are high, large number of packets has to traverse through the forward chain. Thus packet delivery cost increases. So, session arrival and departure rate is also an important issue. WMM uses the concept of forward chain and further takes additional measures to reduce the control overhead and limit the forward chain length. The common problem of the above mentioned schemes is that they are uniform for all the MCs and do not consider the characteristics of an individual MC while performing its mobility management. So, mobility and session activities of each MC need to be considered for mobility management. 

In this paper, a session-to-mobility ratio (SMR) [6] based dynamic mobility management scheme has been proposed. A new SMR calculation scheme is introduced to adapt it in WMN. MC considers both its mobility and the session activity, in the form of SMR, before sending location update to the gateway (GW) and corresponding MRs. Here a threshold SMR value is used, which plays a critical role in the cost of mobility management. Therefore, the optimal threshold SMR value is determined for each individual mesh client dynamically based on the mesh client's specific mobility pattern and session activities.

In this paper an analytical model has been developed to compare the proposed scheme with other baseline schemes such as MEMO, M^3^, and WMM. It has been observed that the proposed scheme outperforms the baseline schemes with respect to total signalling cost. 

The rest of the paper is organized as follows.  Section 2 discusses an overview on relevant mobility management schemes. In Section 3, the SMR based dynamic mobility management scheme has been proposed. The system model and assumptions are discussed in Section 4. The proposed scheme along with some other baseline schemes such as MEMO, M^3^ and WMM are numerically analyzed in Section 5.  Section 6 presents the performance analysis and comparison among the schemes. Finally, Section 7 presents the conclusion and future work.

## 2. Related Work

For the purpose of mobility management in WMN, several techniques have been proposed [7, 8]. In this section, some of the existing strategies such as MEMO [3], M^3^ [4], and WMM [5] have been discussed.

MEsh networks with MObility management (MEMO) [3] use a modified form of AODV (Ad-hoc on-demand distance vector) protocol, called AODV-MEMO, for integrated routing and mobility management. In this scheme, when the MC moves from one MR to another the new MR proactively sends a route reply to the GW to maintain Internet connectivity. On the other hand for maintaining Intranet connectivity it uses a reactive approach. The old MR of the MC floods route error message in the entire network telling other MRs to delete the outdated MC entry from their routing table. On receiving the route error message the corresponding MRs which still need to communicate with the MC transmit route request message for the MC. After receiving the route request message the new MR of MC sends route reply message to the corresponding MRs. The main drawback of this scheme is its signaling overhead due to flooding of route request and route error messages. This signaling overhead becomes much higher if the MNs of the WMN are highly mobile. 

Huang et al. proposed a forward pointer based mobility management scheme named Mesh Mobility Management (M^3^) [4]. In this scheme, the GW keeps track of serving MR for each MC. When the MC moves from one MR to another a forward pointer is added from old MR to new one. MC sends location update message to the GW periodically to update its location information in database of the GW. Thus the forward chain is reset. When the GW receives any Internet packet destined to an MC, it searches for the serving MR of the MC in its database. Then it tunnels the packets to the serving MR of the MC. The serving MR forwards the packet to the MR, within whose vicinity the destination MC currently reside (current MC), through the forward pointer. The uplink Internet packets are sent from current MR of the MC to the GW without tunneling. For Intranet communication, the source MC sends the packets to the GW through its current MR and then GW tunnels the packets to the serving MR of destination MC. The serving MR of destination MC handles the Intranet packet the same way as it does with Internet packets. This scheme decreases location update cost but the drawback with this scheme is its periodic location update procedure which makes the entire scheme very much static. In case of high speed MC, the forward chain length will be large and the packet delivery cost will increase drastically if Internet as well as Intranet traffic to the MC is high. 

Huang et al. proposed a mobility management scheme called Wireless mesh Mobility Management (WMM) [5]. In this scheme, each mesh node (MN) maintains a routing table and a proxy table. The routing table stores the routing paths between the MNs. The proxy table keeps track of other MCs' location information. No separate message is used by the MCs for location update. Instead of that the IP header of each packet carries the location information of source MC. On receiving the packets, intermediate MNs update their proxy table corresponding to the source MC. Thus WMM scheme does not incur any location update cost. When the MC enters into the vicinity of a new MR, the old MR forwards all the packets, destined to MC, routed to it to the new MR. For routing of packets from source MC to destination MC, MRs use their routing and proxy table. If serving MR of source MC does not know the serving MR of the destination MC, it sends all the packets to the GW. The GW checks whether the MC belongs to the WMN or not. If it does not, the packets are considered as Internet packets and are sent to the wired network. Otherwise, the packets are Intranet packets and after receiving the packets, the GW initiates a query procedure by flooding a query message for the destination MC in the entire network. On receiving response from the destination MC, the GW transmits those packets to the destination. The destination MC updates its proxy table and routing table corresponding to the source MC. Now the destination MC can send packets to the source MC directly (not via GW). The drawback of this scheme is its signaling overhead incurred by the query procedure. Moreover, the characteristics of MCs are not considered to achieve the optimal performance. 

The common problem with MEMO, M^3^, and WMM is that the schemes do not consider the characteristics of individual MCs for their mobility management rather they use a static approach which is uniform for all MCs.

## 3. Proposed Scheme

This section presents the proposed mobility management scheme. It uses forward pointer to reduce the number of route update message sent by the MC. To limit the increase in forward chain length, each MC resets the forward chain if its SMR crosses a threshold SMR value. The optimal value for threshold SMR (SMR_oth_) that minimizes the total communication cost per time unit is dynamically determined for each individual MC. The primary objective of this scheme is to minimize the total cost for mobility management. 

In this scheme when the MC joins a WMN, it first gets associated with a nearby MR and sets it as serving MR. Then it sends location update to the GW. The update contains the information about its serving MR. The GW maintains a database recording the serving MRs of all the MCs roaming inside the WMN. On receiving the location update message the GW checks its database whether an entry of the MC is present or not. If there is no entry a new entry of the MC is created. Otherwise, the entry corresponding to the MC is updated. For handling the Intranet traffic each MR maintains a database of the serving MRs of corresponding MCs. There are four major parts in the proposed scheme: calculation of session-to-mobility ratio, calculation of optimal threshold session-to-mobility ratio, mobility management, and routing.

### 3.1. Session-to-Mobility Ratio (SMR) Calculation

In [6] Pack et al. have defined session-to-mobility ratio (SMR) as the ratio of session arrival rate to mobility rate. A session is a stream of consecutive packet at the IP layer. A timer-based approach is used to identify a session [9]. It is similar to the session management technique used in Universal Mobile Telecommunication System (UMTS) [10]. In this technique, each MC will have an active state timer with length *T*_*A*_. If time duration between the receiving of two consecutive packets is greater than *T*_*A*_, the current packet is considered as the first packet of a new session. Otherwise, the packet belongs to the ongoing session. Mobility rate is the MR crossing rate of the MC. In [6] authors have considered only the session arrival rate for computing SMR. But in case of WMN sessions will arrive to as well as depart from the MCs. So, both the factors need to be considered for computing SMR. This technique, considers both session arrival rate to the MC and session departure rate from the MC. The modification in computation of SMR enhances it to capture session and mobility characteristics of the MC more accurately.

### 3.2. Calculation of Optimal Threshold Session-to-Mobility Ratio

SMR_oth_ can be calculated for each individual user using Bat Algorithm (BA) [11]. This is because BA is superior to many other popular optimization algorithms such as Genetic Algorithm (GA) [12–14] and Particle Swarm Optimization (PSO) [15, 16]. From [4] it can clearly observed that BA requires lesser number of function evaluations for a given tolerance or accuracy than that of GA and PSO. Moreover, for a fixed number of function evaluations the accuracy is higher in case of BA.

When the MC enters into the vicinity of new MR it needs to calculate SMR_oth_. But, if the value of SMR_oth_ is calculated after every handoff MC has to perform a lot of computation and it is not feasible for MCs with limited battery power.  Section 6 presents a detailed discussion over the fitness function used. All the components of the fitness function are either constant or average values of variables that are obtained from continuous measurements by the MC. Instantaneous value of any variable has very limited impact on the fitness function. That is why MC can calculate SMR_oth_ value periodically. This periodic calculation reduces the computational work to be carried out by the MC. Thus, battery power consumption rate of the MC is reduced and at the same time it becomes dynamic.

### 3.3. Mobility Management

When the MC moves into the vicinity of a new MR it computes its session-to-mobility ratio (SMR_MC_) and compares it with SMR_oth_. If SMR_MC_ is less than the SMR_oth_, the MC notifies the new MR about its handoff from old MR and also sends the addresses of the corresponding mesh nodes. On receiving the notification, new MR informs the old MR about the handoff and enquires about the serving MR of corresponding mesh nodes of the MC. The old MR replies back by sending addresses of serving MRs of corresponding mesh nodes. A forward pointer is also added from old MR to the new one which is also the current MR of the MC. After receiving the reply from old MR, the new MR updates its database. Thus, the forward chain length of the MC increases by 1. On the other hand, if the SMR_MC_ is greater than or equal to SMR_oth_, same procedure is followed as discussed in the earlier case but no forward chain is added from old MR to new MR, rather the new MR sends location update message to the gateway and the corresponding MRs (if any). When the gateway and corresponding MRs receive the location update message, they search for the entry of the MC in their database and set the current MR as the serving MR of the MC and the forward chain length is reset.

### 3.4. Routing

In the proposed scheme routing of packets is carried out in two different ways depending on the nature of the packet (Internet or Intranet).

#### 3.4.1. Routing of Internet Packets

 Tunneling is used to forward the downstream Internet packets. When the gateway receives a downstream Internet packet, it finds out the serving MR of the destination MC from its database and adds an extra IP header having the address of the serving MR as destination address. This is done because the downstream packets do not have serving MR's address as the destination address and without the serving MR's address intermediate MRs are not able to forward the packet to the serving MR. When the serving MR of the destination MC receives the packet, it decapsulates and if required forwards the packet to current MR through the forward chain. The current MR transmits the packet to the destination MC. 

Tunneling is not used in case of upstream packets. Current MR sends the upstream Internet packets received from the MC through the direct route towards the GW. 

**Figure 1:**
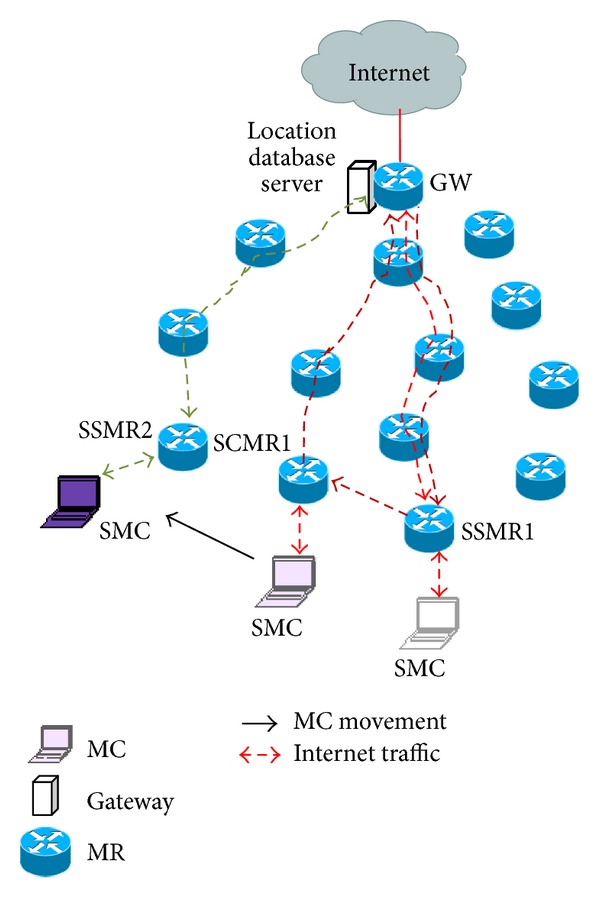
Routing of Internet packet.

Figure 1 shows an example scenario representing routing of Internet traffic. Initially the source MC (SMC) was under the vicinity of source serving MR1 (SSMR1). The SMC sent and received its upstream and downstream Internet packets, respectively, through the SSMR1 to the GW. When the SMC moves from SSMR1 to another MR and gets associated with it, the SMC computes its SMR_MC_ value and compares SMR_oth_ with SMR_MC_. Let its SMR_MC_ value be less than that of SMR_oth_. The SMC does not send location update to the GW rather a forward pointer is added from SSMR1 to new MR named as source current MR1 (SCMR1). When the GW receives a downstream Internet packets destined at the SMC, it encapsulates the packet with a header having address of SSMR1 as destination address. The GW then sends the packets to SSMR1. On receiving, SSMR1 decapsulates the packet and forwards it to SCMR1 through forward chain. Subsequently the SCMR1 delivers it to the SMC. But the upstream packets are sent directly to the GW without tunneling. In this case, SMC sends the upstream packets through the direct path from SCMR1 to the GW. It does not go through SSMR1. After residing some time in SCMR1 the SMC again changes its point of attachment and gets associated with another MR. The SMC computes its SMR_MC_ and compares SMR_MC_ with SMR_oth_. Suppose this time its SMR_MC_ is higher than SMR_oth_. The SMC sends location update message to the GW. This message informs the GW about SMC's association with source serving MR2 (SSMR2). The GW updates SMC's serving MR in its location database. After that all the packets, destined to SMC, are encapsulated by a header having SSMR2's address as destination address and are directly sent to SSMR.

#### 3.4.2. Routing of Intranet Packet

 Intranet traffic routing procedure is almost similar to Internet packet routing. But, in this case packets do not go through the GW. MC sends the upstream packets to its current MR. If the packets are part of a continuing session the current MR adds an extra IP header with each packet and the address of corresponding mesh node's serving MR is set as destination address. After that it sends the packet to the corresponding MR directly. On the other hand, if the packets initiate or respond to a new session, before sending the first packet the current MR sends a query message to the GW to know the serving MR of corresponding MC. After getting reply from the GW the current MR updates its database. Now it can follow the normal packet uplink process. 

Routing of downstream Intranet packets is straight forward. The serving MR of an MC receives the packets destined to it. Then the MR decapsulates and forwards the packets to the current MR of the MC. The routing of Intranet packets by the intermediate MRs is similar to that of Internet traffic.

**Figure 2:**
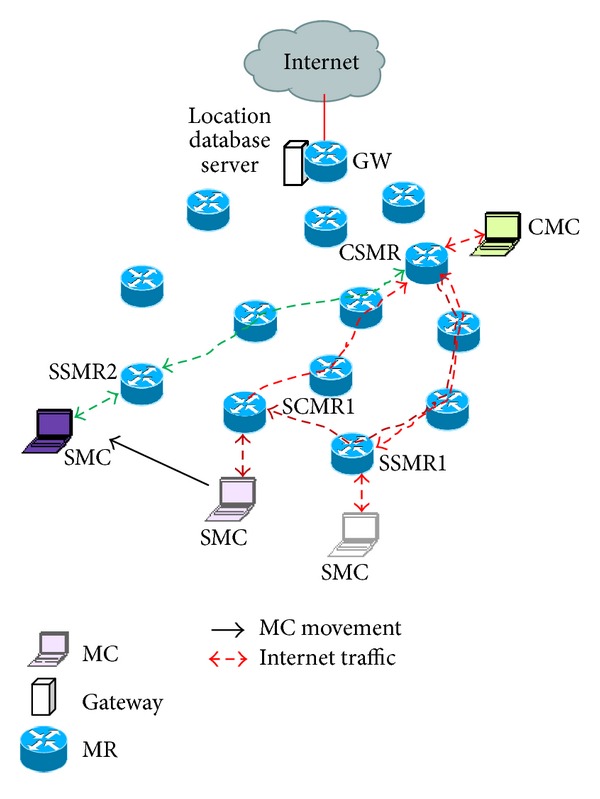
Routing of Intranet packet.

Figure 2 shows an example scenario representing routing of Intranet traffic. Let SMC communicate with corresponding MC (CMC). The scenario is similar to that of Internet shown in Figure 1. Handling of Intranet traffic is also similar. The only difference is that, here CMC is at the other end of communication instead of GW. When SMC is within the vicinity of SSMR1, it sends upstream packets through SSMR1. SSMR1 adds an IP header to those packets. Address of corresponding serving MR (CSMR) of CMC is set as destination address in the IP header. After receiving the packets the CSMR decapsulates and delivers those to CMC. In case of downlink packets towards SMC, the same process is followed. When the SMC moves to SCMR1 the downlink packets for SMC are received by SSMR1 and forwarded to SCMR1. The uplink packets of SMC are directly sent from SCMR1 to CSMR. When the SMC moves from SCMR1 to SSMR2, SSMR2 handles the uplink and downlink packets in the same way as it was in case of SSMR1.

**Figure 3:**
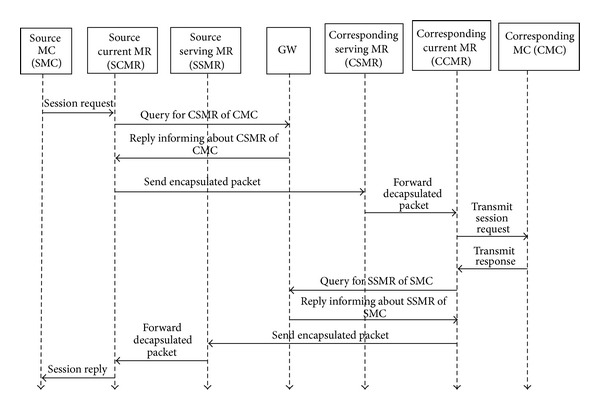
Communication between mesh nodes for session initialization.

Figure 3 shows an example representing message communication among the mesh nodes for new session initialization between SMC and CMC. At first SMC sends session request to SCMR. After receiving the session initialization request the SCMR sends a query message to the GW for the address of CSMR of CMR. The GW sends reply to SCMR informing it about CSMR of CMC. Then SCMR sends encapsulated packet to CSMR. CSMR decapsulates the packet and forwards it to corresponding current MR (CCMR). CCMR delivers the packet to CMC. On receiving the request CMC sends response message to CCMR. Again CCMR searches for the SSMR of SMC as SCMR has done for CMR. After that CCMR sends encapsulated packet to SSMR of SMC. SSMR decapsulates the packet and forwards it to SMC through SCMR.

## 4. System Model and Assumptions

This section describes the system model and assumptions. Without loss of generality the following assumptions can be made.

- Incoming and outgoing sessions at an MC occur according to Poisson process with parameter *λ*_*a*_ and *λ*_*d*_, respectively [17].
- Residence time (*t*_*s*_) of the MC in the MR follows an exponential distribution with parameter *λ*_*s*_ [18].

The number of occurrences of an event within a time unit follows a Poisson distribution with parameter *λ* if and only if the time elapsed between two consecutive occurrences of the event has an exponential distribution with parameter *λ* and it is independent of previous occurrences [19]. It has been assumed that the session arrival and departure rate follow Poisson process with rates *λ*_*a*_, and *λ*_*d*_, respectively, and arrival and departure of a session are independent of previous sessions. So, intersession arrival time (*t*_*a*_) and departure time (*t*_*d*_) follow exponential distribution with parameters *λ*_*a*_ and *λ*_*d*_, respectively. Similarly, the number of associations of an MC with MRs in a time unit follows Poisson distribution with rate (mobility rate) *λ*_*s*_.

In addition to the above mentioned assumptions some parameters are also used for numerical analysis of the proposed scheme and comparison with other baseline schemes.  Table 1 shows the parameters used for mathematical modeling and their interpretations.

## 5. Numerical Analysis

This section presents a numerical analysis of the proposed SMR based scheme, MEMO, M^3^, and WMM. Based on the assumptions and system model described in the previous section total communication cost/time unit for each of the schemes has been calculated. Handoff cost, packet delivery cost and query cost per time unit together form the total communication cost/time unit. 

Considering both session arrival and session departure SMR_MC_ value can be calculated as

(1) $$\begin{array}{c} {\text{SMR}}_{\text{MC}}\;=\frac{t_{s}}{\left(t_{a}+t_{d}\right)}. \end{array}$$

Probability that SMR_MC_ value be smaller than *δ*_th_ is calculated as

(2) Pth=  P  (SMRMC<  δth)=P(ts(ta+td)<δth)    =P(ts<δth(ta+td)).

Let *t*_*a*_ = *t*_1_,   *t*_*d*_ = *t*_2_, *λ*_*a*_   = *λ*_1_ and *λ*_*d*_ = *λ*_2_. So, *t*_*a*_ + *t*_*d*_ = *t*_1_ + *t*_2_. Let *t*_1_ + *t*_2_ = *S*_2_. The probability density function of *t*_1_ and *t*_2_ for *t* ≥ 0 is given as

(3) $$\begin{array}{c} f_{t_{a}}\left(t\right)=f_{t_{1}}\left(t\right)=\lambda_{1}e^{-t\lambda_{1}},\\ f_{t_{d}}\left(t\right)=f_{t_{2}}\left(t\right)=\lambda_{2}e^{-t\lambda_{2}}. \end{array}$$

If *λ*_1_ ≠ *λ*_2_, from the theorem of convolution of exponential distribution with different parameters, presented in [20], and (3), probability density function of *S*_2_ can be calculated as

(4) $$\begin{array}{c} f_{s_{2}}\left(t\right)=\sum_{i=1}^{2}\left(\frac{\lambda_{1}\lambda_{2}}{\prod_{\begin{array}{c} j\;=\;1\\ i\;\neq \;j \end{array}}^{2}\left(\lambda_{j}-\lambda_{i}\right)}e^{-t\lambda_{i}}\right). \end{array}$$

Using (4) on (2) *P*_th_ can be calculated as

(5) Pth=P(SMRMC<δth)=∫0∞[P(ts<δthτ).∑i=12(λ1λ2∏j = 1i ≠ j2(λj−λi)e−τλi)]dτ=∫0∞[P(ts<δthτ)   .{λ1λ2(λ2−λ1)e−τλ1+λ1λ2(λ1−λ2)e−τλ2}]dτ=λ1λ2(λ2−λ1)[{∫0∞P(ts<δthτ)e−τλ1dτ}       −{∫0∞P(ts<δthτ)e−τλ2dτ}].

If *δ*_th_ · *τ* = *y*, (5) becomes

(6) =λ1λ2δth(λ2−λ1)[{∫0∞P(ts<y)e−yλ1/δthdy}        −{∫0∞P(ts<y)e−yλ2/δthdy}],∫0∞P(ts<y)e−yλ1/δthdy  =∫0∞(∫0yλse−λsxdx)e−yλ1/δthdy  =δth2λsλ1(δthλs+λ1).

Similarly,

(7) $$\begin{array}{c} \int_{0}^{\infty }P\left(t_{s}<y\right)e^{-y\lambda_{2}/\delta_{\text{th}}}dy=\frac{\delta_{\text{th}}^{2}\lambda_{s}}{\lambda_{2}\left(\delta_{\text{th}}\lambda_{s}+\lambda_{2}\right)}. \end{array}$$

From (5), (6), and (7) the value of *P*_th_ can be computed as

(8) Pth=P(SMRMC<δth)=λ1λ2λsδthλ2−λ1[1λ1(δthλs+λ1)−1λ2(δthλs+λ2)]=λaλdλsδthλd−λa[1λa(δthλs+λa)−1λd(δthλs+λd)].

If *λ*_1_ = *λ*_2_ = *λ*, from the theorem of convolution of exponential distribution with same parameter [20, 21] and (3) it can be written that *S*_2_ follows gamma distribution with parameter (2, *λ*) and has probability distribution function:

(9) $$\begin{array}{c} f_{s_{2}}\left(t\right)=\lambda^{2}te^{-\lambda t}. \end{array}$$

Using (9) on (2) *P*_th_ can be calculated as

(10) $$\begin{array}{c} P_{\text{th}}=P\left({\text{SMR}}_{\text{MC}}<\delta_{\text{th}}\right)=\int_{0}^{\infty }\left[P\left(t_{s}<\delta_{\text{th}}\tau \right)\lambda^{2}e^{-\lambda \tau }\tau \right]d\tau . \end{array}$$

If *δ*_th_ · *τ* = *y*, (10) becomes

(11) =λ2δth∫0∞{P(ts<y)e−(λy/δth)yδth}dy=λ2δth∫0∞{(1−eλst)e−(λy/δth)yδth}dy=1−λs2(λsδth+λ)2.

From (8) and (11) it can be written that

(12) $$\begin{array}{c} P_{\text{th}}=\{\begin{array}{cc} \frac{\lambda_{a}\lambda_{d}\lambda_{s}\delta_{\text{th}}}{\lambda_{d}-\lambda_{a}} & \\ \;\times [\frac{1}{\lambda_{a}\left(\delta_{\text{th}}\lambda_{s}+\lambda_{a}\right)} & \\ \;\;\;-\frac{1}{\lambda_{d}\left(\delta_{\text{th}}\lambda_{s}+\lambda_{d}\right)}] & \text{if}\;\lambda_{a}\neq \lambda_{d}\\ 1-\frac{\lambda_{s}^{2}}{{\left(\lambda_{s}\delta_{\text{th}}+\lambda \right)}^{2}} & \text{if}\;\lambda_{a}=\lambda_{d}=\lambda . \end{array} \end{array}$$

### 5.1. Handoff Cost

In MEMO, when the MC moves from one MR to another the old MR broadcasts route error message informing other MRs that the MC has moved out of its vicinity. Since each MR broadcasts the route error message, cost of broadcasting the message is calculated as *M*. The new MR proactively sends route reply message to the GW. The cost for this route reply is *α* × *γ*. After Handoff, if the MC wants to continue the communication with any corresponding MC, the new MR broadcast route requests message to all the MRs for searching the corresponding MC. It has the cost of *M*. The host MR of corresponding MC sends back route reply message costing *β* × *γ*. If the corresponding MC wants to continue, its host MR performs the same procedure and the new MR sends back route reply message. Since there are *N*_active_ number of corresponding MCs, the cost incurred in this process is {*N*_active_ × (*M* + *β* × *γ*)}. So, for MEMO handoff cost in a time unit is calculated as

(13) $$\begin{array}{c} C_{\text{hMEMO}}=\left\{M+\alpha \times \gamma +N_{\text{active}}\times \left(M+\beta \times \gamma \right)\right\}\times \lambda_{s}. \end{array}$$

In M^3^, the MC sends location update message to the GW periodically once in every *t*_M^3^_ time units. Otherwise, a forward pointer is added from old MR to new MR. So, for M^3^ the handoff cost in a time unit is calculated as

(14) $$\begin{array}{c} C_{\text{h}{\text{M}}^{3}}=\left\{2\times \gamma \times \frac{t_{{\text{M}}^{3}}-1}{t_{{\text{M}}^{3}}}+\alpha \times \gamma \times \frac{1}{t_{{\text{M}}^{3}}}\right\}\times \lambda_{s}. \end{array}$$

In WMM the no separate location update message is used but location update is done through the packets sent from source MC. Only a forward pointer is added from old MR to new MR. Handoff cost in a time unit for WMM can be computed as

(15) $$\begin{array}{c} C_{\text{hWMM}}=2\times \gamma \times \lambda_{s}. \end{array}$$

In SMR based scheme, when the MC moves form vicinity of one MR to another, the MC calculates its SMR_MC_ value, compares it with *δ*_th_, and performs the handoff process. If SMR_MC_ is less than *δ*_th_ a forward pointer is added from old MR to new MR. Otherwise, new MR sends location update message to the GW as well as all corresponding MRs. So, the handoff cost can be calculated as

(16) $$\begin{array}{c} C_{\text{hSMR}}\;=\{\begin{array}{cc} 2\cdot \gamma & \text{if}\;{\text{SMR}}_{\text{MC}}<\delta_{\text{th}}\\ \left(\alpha +N_{\text{active}}\cdot \beta \right)\cdot \gamma & \text{if}\;{\text{SMR}}_{\text{MC}}\geq \delta_{\text{th}}. \end{array} \end{array}$$

The handoff cost in a time unit will be

(17) ChSMR={2×γ×P(SMRMC<δth)+(α+Nactive×β)  ×  γ×P(SMRMC≥δth)}×λs={2×γ×Pth+(α+Nactive×β)×γ×Pth}×λs.

### 5.2. Packet Delivery Cost

As the MC roams within the WMN with respect to a starting reference MR, there are two possibilities when it moves from one MR to another. The MC can move to a previously visited MR or a new MR. If it moves to a previously visited MR, the displacement (hop count) from the reference MR decreases. Otherwise, the displacement increases by 1. The displacement depends on the topology of the WMN and also the mobility pattern of the MC. When the MC moves form one MR to another and gets associated with it, calculation of MN's displacement from the reference MR is very complicated. For simplicity it has been assumed that average displacement (with respect to hop count) of the MC per MR association is *c* [22]. For example, let a MC move from MR A to MR B. While moving from A to B the MC gets through *l* number of association with MRs and the distance (hop count) between A and B is *k*. So, from the assumption it can be written that *k* = *cl*.

Packet delivery cost incurred by MEMO in a time unit (*C*_pMEMO_) consists of downlink Internet packet delivery cost (*C*_pinterdMEMO_), uplink Internet packet delivery cost (*C*_pinteruMEMO_), and Intranet packet delivery cost (*C*_pintraMEMO_):

(18) CpMEMO=CpinterdMEMO×λp×λa×Ia ×rinter100+CpinteruMEMO×λp×λa ×Ia×(1−rinter100)+CpinterdMEMO×λp ×λd×Id×rinter100+CpinteruMEMO×λp ×λd×Id×(1−rinter100)+CpintraMEMO ×λp×λa×(1−Ia)+CpintraMEMO ×λp×λd×(1−Id).

In MEMO the downlink Internet packets of MC are directly sent from the GW to host MR. The host MR then transmits it to MC. On the other hand, in case of uplink packets the reverse procedure is followed. So,

(19) $$\begin{array}{c} C_{\text{pinterdMEMO}}=C_{\text{pinteruMEMO}}=\alpha \cdot \gamma . \end{array}$$

For Intranet communication the host MR of source MC transmits and receives the packets to and from the host MR of destination MC directly. Thus, Intranet packet delivery cost can be calculated as,

(20) $$\begin{array}{c} C_{\text{pintraMEMO}}=\beta \cdot \gamma . \end{array}$$

Packet delivery cost/time unit incurred by M^3^ in a time unit (*C*_pM^3^_) consists of downlink Internet packet delivery cost (*C*_pinterdM^3^_), uplink Internet packet delivery cost (*C*_pinteruM^3^_), downlink Intranet packet delivery cost (*C*_pintradM^3^_), and uplink Intranet packet delivery cost (*C*_pintrauM^3^_):

(21) CpM3=λa×λp×Ia ×(CpinterdM3×rinter100+CpinteruM3×(1−rinter100)) +λd×λp×Id ×(CpinterdM3×rinter100+CpinteruM3×(1−rinter100)) +λa×λp×(1−Ia) ×(CpintradM3×rintra100+CpintrauM3×(1−rintra100)) +λd×λp×(1−Id) ×(CpintradM3×rintra100+CpintrauM3×(1−rintra100)).

The cost of transferring the downlink Internet packets from GW to the serving MR of the MC is *α* × *γ*. The serving MR forwards the packets to the current MR through forward chain. Since average displacement of the MC per MR association is *c*, the average chain length is (*c* × *t*_M^3^_ × *λ*_*s*_)/2. Thus, the downlink Internet packet delivery cost is

(22) $$\begin{array}{c} C_{\text{pinterd}{\text{M}}^{3}}=\left(\alpha +\frac{\left(c\times t_{{\text{M}}^{3}}\times \lambda_{s}\right)}{2}\right)\times \gamma . \end{array}$$

The uplink Internet packets are directly sent from current MR to the GW without tunneling. The cost for uplink Internet packet is

(23) $$\begin{array}{c} C_{\text{pinteru}{\text{M}}^{3}}=\alpha \times \gamma . \end{array}$$

At first the downlink Intranet packets are sent from the corresponding MC to the GW incurring cost of *α* × *γ*. The GW then sends the packets to the MC the same way it did with downlink Internet packets which costs {(*α* +( *c* × *t*_M^3^_ × *λ*_*s*_)/2) × *γ*}. So, downlink packet delivery cost can be calculated as

(24) $$\begin{array}{c} C_{\text{pintrad}{\text{M}}^{3}}=\left(2\times \alpha +\frac{\left(c\times t_{{\text{M}}^{3}}\times \lambda_{s}\right)}{2}\right)\times \gamma . \end{array}$$

Routing of uplink Intranet packets from current MR of MC to GW has cost of *α* × *γ*. Then tunneling and followed by forwarding of those packets to the destination MC cost {(*α* + (*c* × *t*_M^3^_ × *λ*_*sc*_)/2) × *γ*}. So, uplink packet delivery cost can be calculated as

(25) $$\begin{array}{c} C_{\text{pintrau}{\text{M}}^{3}}=\left(2\times \alpha +\frac{\left(c\times t_{{\text{M}}^{3}}\times \lambda_{sc}\right)}{2}\right)\times \gamma . \end{array}$$

In WMM, packet delivery cost per time unit (*C*_pWMM_) consists of downlink Internet packet delivery cost (*C*_pinterdWMM_), uplink Internet packet delivery cost (*C*_pinteruWMM_), cost for downlink Intranet packet delivery through GW (*C*_pintragdWMM_), downstream direct Intranet packet delivery cost (*C*_pintradWMM_), cost for upstream Intranet packet delivery through GW (*C*_pintraguWMM_), and upstream direct Intranet packet delivery cost (*C*_pintrauWMM_):

(26) CpWMM=CpinterdWMM×λp×λa×Ia×rinter100 +CpinteruWMM×λp×λa×Ia×(1−rinter100) +CpinterdWMM×λp×λd×Id×rinter100 +CpinteruWMM×λp×λd×Id×(1−rinter100) +CpintragdWMM×λp×λa×(1−Ia)×pg×rintra100 +CpintradWMM×λp×λa×(1−Ia)×(1−pg) ×rintra100+CpintragdWMM×λp×λd ×(1−Id)×pg×rintra100 +CpintradWMM×λp×λd×(1−Id)×(1−pg) ×rintra100+CpintraguWMM×λp×λa ×(1−Ia)×pg×(1−rintra100) +CpintrauWMM×λp×λa×(1−Ia)×(1−pg) ×(1−rintra100)+CpintraguWMM×λp×λd ×(1−Id)×pg×(1−rintra100) +CpintrauWMM×λp×λd×(1−Id) ×(1−pg)×(1−rintra100).

In WMM, MC sends its location information with every packet (Internet or Intranet). Between the arrivals of two consecutive packets at an MR, the movement of source MC may result in a number of handoffs and a chain of proxy table entries may be formed. Such chain is similar to forward pointer. The chain associated with source MC gets reset with the arrival of packet from it to the MR. Average time interval between such chain reset operations is the same as the interarrival time of two consecutive packets.

 Average rate of Internet packet arrival at the GW from a source MC is

(27) ARinterg=λa×λp×Ia×(1−(rinter100)) +λd×λp×Id×(1−(rinter100)).

On the other hand, average Intranet packet arrival rate at the GW from the source MC is

(28) ARintrag=λa×λp×(1−Ia)×pg ×(1−rintra100)+λd×λp ×(1−Id)×pg×(1−rintra100).

The summation of Internet and Intranet packet arrival rate at the GW from the MC will together form the total packet arrival rate. The interarrival time of two consecutive packets is

(29) $$\begin{array}{c} T_{\text{iag}}=\frac{1}{\text{A}{\text{R}}_{\text{interg}}+\text{A}{\text{R}}_{\text{intrag}}}. \end{array}$$

The average distance an MC can move between two consecutive forward chain reset operations and can be computed as

(30) $$\begin{array}{c} \text{F}{\text{C}}_{\text{gmWMM}}=T_{\text{iag}}\times \lambda_{s}\times c. \end{array}$$

So, the downlink Internet packet delivery cost is

(31) $$\begin{array}{c} C_{\text{pinterdWMM}}=\left(\alpha +\text{F}{\text{C}}_{\text{gmWMM}}\right)\times \gamma . \end{array}$$

Since uplink packets are sent to the GW directly from the current MC of the MR without using any proxy table chain entries, uplink Internet packet delivery cost is

(32) $$\begin{array}{c} C_{\text{pinteruWMM}}=\alpha \times \gamma . \end{array}$$

The downstream Intranet packets that are sent through the GW will first traverse from the corresponding MC to the GW. This is similar to uplink of Internet packets. Then GW sends those packets to the MC through the forward chain, as it does with downstream Internet packets. So, cost for downlink Intranet packet delivery through GW is

(33) $$\begin{array}{c} C_{\text{pintragdWMM}}=\left(2\times \alpha +\text{F}{\text{C}}_{\text{gmWMM}}\right). \end{array}$$

The forward chain length for downstream Internet communication of corresponding MC can be computed by following the same process discussed above. Only *λ*_*s*_, *λ*_*a*_, and *λ*_*d*_ will be replaced by *λ*_sc_, *λ*_ac_, and *λ*_dc_, respectively. So the forward chain is

(34) FCgmWMMc=(λsc×c) ×(λac×λp×Ia×(1−rinter100)  +λdc×λp×Id×(1−rinter100)  +λac×λp×(1−Ia)×pg  ×(1−rintra100)+λdc×λp  ×(1−Id)×pg  ×(1−rintra100))−1.

The routing of upstream Intranet packets through GW is the same as downstream Intranet packets through GW. The only difference is that the source MC will directly send the packets to the GW and forward chain will be used at the end of corresponding MC. So, cost for upstream Intranet packet delivery through GW is

(35) $$\begin{array}{c} C_{\text{pintraguWMM}}=\left(2\times \alpha +\text{F}{\text{C}}_{\text{gmWMM}}^{c}\right). \end{array}$$

The effective arrival rate of downstream Intranet packets originated from a corresponding MC reaching the MC is

(36) $$\begin{array}{c} \text{A}{\text{R}}_{\text{intrad}}=\frac{\left\{\lambda_{p}\times \lambda_{a}\times \left(1-I_{a}\right)+\lambda_{d}\times \lambda_{p}\times \left(1-I_{d}\right)\right\}\times r_{\text{intra}}}{N_{\text{active}}\times 100}. \end{array}$$

Average interarrival time between two such consecutive packet is

(37) $$\begin{array}{c} T_{\text{intrad}}=\frac{1}{\text{A}{\text{R}}_{\text{intrad}}}. \end{array}$$

The average distance that the corresponding MC can move within this time interval is

(38) $$\begin{array}{c} \text{F}{\text{C}}_{\text{intraWMM}}^{c}=T_{\text{intrad}}\times \lambda_{\text{sc}}\times c. \end{array}$$

Delivery cost of upstream Intranet packets from source MC to corresponding MC through the forward chain is

(39) $$\begin{array}{c} C_{\text{pintrauWMM}}=\left(\beta +\text{F}{\text{C}}_{\text{intraWMM}}^{c}\right)\times \gamma . \end{array}$$

The effective arrival rate of upstream Intranet packets of source MC reaching a corresponding MC is

(40) ARintrau ={λp×λa×(1−Ia)+λd×λp×(1−Id)}×(1−rintra)Nactive×100.

In this case average interarrival time is

(41) $$\begin{array}{c} T_{\text{intrau}}=\frac{1}{\text{A}{\text{R}}_{\text{intrau}}}. \end{array}$$

The average distance that the source MC can move within this time interval is

(42) $$\begin{array}{c} \text{F}{\text{C}}_{\text{intraWMM}}=T_{\text{intrau}}\times \lambda_{s}\times c. \end{array}$$

Delivery cost of downstream Intranet packets from corresponding MC to source MC through the forward chain is

(43) $$\begin{array}{c} C_{\text{pintradWMM}}=\left(\beta +\text{F}{\text{C}}_{\text{intrawmm}}\right)\times \gamma . \end{array}$$

In the proposed scheme, when the MC moves to a new MR with respect to the forward chain from serving MR and its SMR_MC_ < *δ*_th_, the forward chain length will increase by 1. But if it moves to any MR in the forward chain, the chain length will decrease. Since average displacement of the MC per MR association is *c*, it can be written that, when the MC moves from one MR to another and its SMR_MC_ < *δ*_th_ the forward chain length will increase by *c*. The upper bound for the forward chain length is (*M* − 1) and the required number of MR association is ((*M* − 1)/*c*). 

In this scheme packet delivery cost in a time unit (*C*_pSMR_) consists of downstream Internet packet delivery cost (*C*_pinterdSMR_), upstream Internet packet delivery cost (*C*_pinteruSMR_), downstream Intranet packet delivery cost (*C*_pintradSMR_), and upstream Intranet packet delivery cost (*C*_pintrauSMR_):

(44) CpSMR=CpinterdSMR×λp×λa×Ia×rinter100 +CpinteruSMR×λp×λa×Ia×(1−rinter100) +CpinterdSMR×λp×λd×Id×rinter100 +CpinteruSMR×λp×λd×Id×(1−rinter100) +CpintradSMR×λp×λa×(1−Ia)×rintra100 +CpintrauSMR×λp×λa×(1−Ia)×(1−rintra100) +CpintradSMR×λp×λd×(1−Id)×rintra100 +CpinteruSMR×λp×λd×(1−Id)×(1−rintra100).

The GW will send the downlink Internet packets to the serving MR and the serving MR will forward these packets to the MC through the forward chain. Let *P*(*i*) be the probability that forward chain length of the MC is *i* × *c*  and *C*_pinterdSMR_(*i*) be the downstream Internet packet delivery cost with that forward chain length. The cost per downstream packet delivery can be calculated as

(45) CpinterdSMR  =∑i=0(M−1)/cCpinterdSMR(i)·P(i)=  (1−Pth)·α·γ+(1−Pth)·Pth·(α+c)·γ +(1−Pth)·Pth2·(α+2c)·γ+⋯+(1−Pth) ·Pth((M−1)/c)−1·(1−Pth) ·(α+(M−1c−1)·c) ·γ  +Pth(M−1)/c  ·(α+(M−1))·γ=  α·γ+γ·c·Pth·[1−Pth((M−1)/c)(1−Pth)].

As a check it can be noted that

(46) ∑i=0(M−1)/cP(i)=P(⋃i=0((M−1)/c)−1N=i)+P(M−1)/c=((1−Pth)·∑i=0((M−1)/c)−1Pthi)+Pth(M−1)/c=1.

The upstream packets from the MC will not be tunneled by serving MR rather will directly be sent to the GW. So, cost per upstream packet delivery is

(47) $$\begin{array}{c} C_{\text{pinteruSMR}}=\;\alpha \cdot \gamma . \end{array}$$

Current MR of corresponding MC will tunnel downstream Intranet packets of MC to its serving MR through *β* number MRs. Then serving MR forwards the packets to MC through forward chain. So, calculation of downstream Intranet packet delivery cost is similar to that of downstream Internet packets:

(48) CpintradSMR  =∑i=0(M−1)/cCpintradSMR(i)·P(i)  =  (1−Pth)·β·γ+(1−Pth)·Pth·(β+c)·γ +(1−Pth)·Pth2·(β+2c)·γ +⋯+(1−Pth)·Pth((M−1)/c)−1·(1−Pth) ·(β+(M−1c−1)·c)·γ  +  Pth(M−1)/c ·(β+(M−1))·γ=  β·γ+γ·c·Pth·[1−pth((M−1)/c)(1−Pth)].

In case of corresponding MCs, let the probability that its SMR value is smaller than *δ*_thc_ be *P*_thc_. Calculation of *P*_thc_ is similar to (8)

(49) Pthc=λacλdcλscδthcλdc−λac ×[1λac(δthcλsc+λac)−1λdc(δthcλsc+λdc)].

Upstream Intranet packets are tunneled from the current MR of source MC to serving MR of destination MC. Then the serving MR of corresponding MC forwards the packets to it. The cost for upstream Intranet packets is

(50) $$\begin{array}{c} C_{\text{pintrauSMR}}\;=\;\beta \cdot \gamma +\gamma \cdot c\cdot P_{\text{thc}}\cdot \left[\frac{1-p_{\text{thc}}^{\left(\left(M-1\right)/c\right)}}{\left(1-P_{\text{thc}}\right)}\right]. \end{array}$$

### 5.3. Query Cost

In MEMO, when the source MC sends request to its host MR for initialization of Intranet session and if the host MR does not have a route to the destination MC it broadcasts a route request message which costs *P*_*r*_ × *M*. On receiving the route request the host MR of destination MC sends back route reply costing *β* × *γ*. So, query cost for MEMO in a time unit is

(51) $$\begin{array}{c} C_{\text{qMEMO}}\;=\;\left(P_{r}\times M+\;\beta \times \gamma \right)\times \left(1-I_{d}\right)\times \lambda_{d}\times P_{g}. \end{array}$$

In M^3^, MC does not send any query message for Internet or Intranet communication because all the packets are sent through the GW.

In WMM, GW will execute location query procedure only when it has packets to send to an MC before the MC initiates the first Internet session, after the MC joins a WMN or wakes up and reconnects to the WMN after staying in sleep mode for some time. Let *ω*_*w*_ and *ω*_*s*_ be switching rate from sleep mode to active mode and the reverse mode switching rate, respectively. As discussed in [5] the reconnection rate of MC is

(52) $$\begin{array}{c} \omega =\frac{1}{1/\omega_{w}\;+1/\omega_{s}\;}=\frac{\omega_{w}\times \omega_{s}}{\omega_{s}+\omega_{s}}. \end{array}$$

For searching the MC, GW broadcasts route request message to all the MRs of the WMN which costs *P*_*r*_ × *M*. The serving MR of corresponding MC sends back response message costing *α* × *γ*. So, query cost in a time unit is

(53) $$\begin{array}{c} C_{\text{qWMM}}=\left(P_{r}\times M+\alpha \times \gamma \right)\times P_{q}\times \omega . \end{array}$$

As Figure 3 shows, in the proposed scheme for initialization of every departing session, the current MR of source MC sends query message to the GW for locating destination MR. The GW sends back the information of serving MR of destination MC. From the end of destination MC, the current MR of destination MC will also send such query message to the GW for sending the reply packets to source MC. These four message transfers will cost 4 × *α* × *γ*. The query cost for this scheme in a time unit is

(54) $$\begin{array}{c} C_{\text{qSMR}}=\;4\times \alpha \times \gamma \times \lambda_{d}. \end{array}$$

### 5.4. Total Communication Cost

Total communication cost in a time unit for MEMO, M^3^, WMM, and the proposed SMR based scheme is denoted by TC_MEMO_, TC_M^3^_, TC_WMM_, and TC_SMR_, respectively. So, it can be written that

(55) $$\begin{array}{c} {\text{TC}}_{\text{MEMO}}=C_{\text{hMEMO}}+C_{\text{pMEMO}}+C_{\text{qmemo}},\\ {\text{TC}}_{{\text{M}}^{3}}=C_{\text{h}{\text{M}}^{3}}+C_{\text{p}{\text{M}}^{3}},\\ {\text{TC}}_{\text{WMM}}=C_{\text{hWMM}}+C_{\text{pWMM}}+C_{\text{qWMM}},\\ {\text{TC}}_{\text{SMR}}=C_{\text{hSMR}}+C_{\text{pSMR}}+C_{\text{qSMR}}. \end{array}$$

## 6. Fitness Function for Optimization

Each MC will periodically compute optimal threshold SMR value SMR_oth_ using bat algorithm and will be set as *δ*_th_. The objective of finding the SMR_oth_ is to minimize total communication cost/time unit (TC_SMR_). The fitness function for the objective can be obtained by using (17), (44), (45), (47), (48), (50), and (55). So, the fitness function is

(56) f(δth)={2·γ·Pth+(α+Nactive·β)·γ·Pth}  ·λs+γ·c·Pth·λp100(1−Pth((M−1)/c)1−Pth) ·[rinter(λa·Ia+λd·Id)   +  rintra(λa·(1−Ia)+λd·(1−Id))].

## 7. Performance Analysis

In this section, the performances of MEMO, M^3^, and WMM are compared with the proposed scheme in terms of handoff cost, packet delivery cost, and total communication cost per time unit under different mobility rate, session arrival rate, and session departure rate. To compare the cost incurred by any of the three schemes (*C*_other_) with that of proposed SMR based scheme (*C*_SMR_) the following metric is used

(57) $$\begin{array}{c} F\%=\;\left(\frac{\left|C_{\text{other}}-C_{\text{SMR}}\right|}{C_{\text{SMR}}}\right)\times 100. \end{array}$$

If *C*_other_ > *C*_SMR_, it can be said that the cost incurred by the other schemes is *F*% higher compared to the SMR based scheme. Otherwise, the cost of the other scheme is *F*% less compared to the SMR based scheme. The variation in the value of SMR_oth_ with the change in *λ*_*s*_, *λ*_*a*_, and *λ*_*d*_ is also analyzed.  Table 2 shows the default values of the parameters used in the numerical analysis. Here second is considered as time unit. To find the optimal threshold SMR value (SMR_oth_) for minimum total communication cost/time unit bat algorithm is used with population size 100, number of generation 50, loudness 100, and pulse rate 0.5.

**Figure 4:**
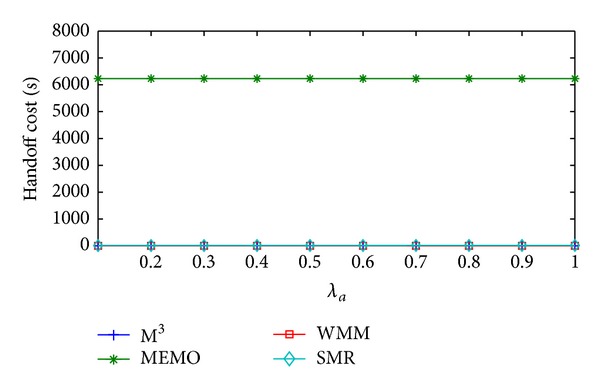
Handoff cost/sec versus *λ*_*a*_.

Figure 4 shows the variation in handoff cost/sec of MEMO, M^3^, WMM, and the proposed SMR based scheme with respect to the increase in *λ*_*a*_. In this case, the values of *λ*_*s*_ and *λ*_*d*_ are assumed to be 0.2 and 0.5, respectively. In MEMO after every handoff the old MR of MC broadcasts route error message in the entire network and new MR sends route reply message to the GW proactively. Thus it incurs high handoff cost/sec. In M^3^ location update messages are sent by the MC to the GW after a static time interval. Therefore, the MC has to send location update message less frequently and handoff cost/sec becomes less compared to MEMO. In case of WMM, MC does not send any location update message rather location information is carried by each data packet originated from the MC. So, the handoff cost/sec is the lowest among all other schemes. In the proposed scheme, the MC sends location update message to the GW and the host MRs of corresponding MCs only when its SMR is greater than or equal to *δ*_th_. This leads to less frequent location updates. Handoff cost/sec of MEMO, M^3^, and WMM is constant even if *λ*_*a*_ increases. This is because the frequency of sending location update message of the three schemes remains unchanged with the increase in *λ*_*a*_. On the other hand, in SMR based scheme, as *λ*_*a*_ increases SMR value of MC increases and frequency of SMR value crossing *δ*_th_ also increases. Thus handoff cost/sec increases with the increase in *λ*_*a*_ but the rate of increase is very little. Therefore, average handoff cost/sec of MEMO is 45,588.35% higher compared to the proposed SMR based scheme. On the other hand, compared to the SMR based scheme M^3^ and WMM incur 99.70% and 99.71% less average handoff cost/sec, respectively.

**Figure 5:**
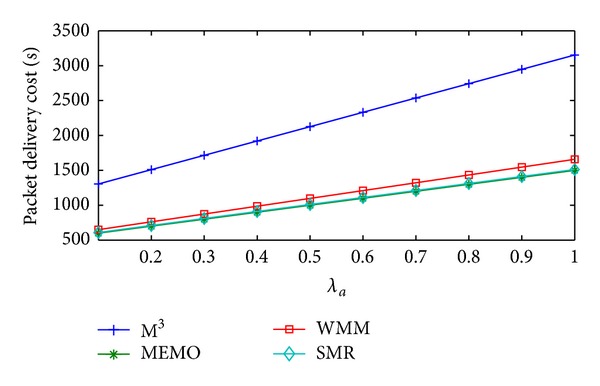
Packet delivery cost/sec versus *λ*_*a*_.

Figure 5 shows the change in packet delivery cost/sec of MEMO, M^3^, WMM, and SMR based scheme with respect to the increase in *λ*_*a*_. MEMO has the lowest packet delivery cost/sec since packets are directly delivered to the current MR of MC and no chain for packet forwarding is used. In M^3^, the packets are forwarded through long forward chain. The forward chain length keeps growing and gets reset after some fixed time interval. This results in the highest packet delivery cost/sec in M^3^. In WMM a chain of proxy table entries is formed due to the mobility of MC within the time interval between two consecutive packet arrivals originated from the MC to the GW or corresponding MC. The packets have to go through the forward chain which is of significant length. In the proposed SMR based scheme because of the selection of optimal threshold SMR value packets have to traverse through the forward chain of optimal length. The packet delivery cost/sec of the MEMO increases with increase in *λ*_*a*_ but it is due to the increased number of packet transfer/sec. As *λ*_*a*_ increases, packet delivery cost/sec of M^3^ also increases because more numbers of packets are forwarded through the long forward chain. In WMM because of increase in *λ*_*a*_ though the forward chain resets will be frequent but more number of packets has to go through the forward chain. That is why the packet delivery cost/sec increases as *λ*_*a*_ increases. In SMR based scheme *λ*_*a*_ is incorporated in the calculation of SMR and optimal value for *δ*_th_ is selected dynamically. As a result the packet delivery cost/sec remains close to that of MEMO with the increase in *λ*_*a*_. Therefore, compared to SMR based scheme M^3^ and WMM have 110.16% and 8.8% higher average packet delivery cost/sec, respectively. On the other hand, MEMO incurs 0.99% less average packet delivery cost/sec compared to the SMR based scheme.

**Figure 6:**
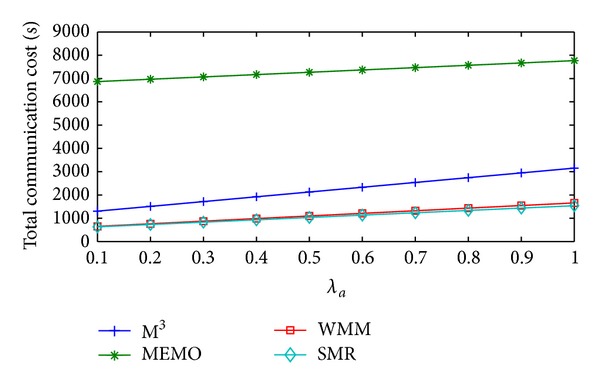
Total communication cost/sec versus *λ*_*a*_.

Figure 6 shows the change in total communication cost/sec of MEMO, M^3^, WMM, and SMR based scheme with respect to the increase in *λ*_*a*_. MEMO has the highest total communication cost/sec. This is due to its huge location update cost/sec and significant query cost/sec. Total communication cost/sec of M^3^ is lower than MEMO because M^3^ incurs much lesser handoff cost/sec than MEMO. Handoff cost/sec of WMM is the minimum but it incurs a significant amount of packet delivery cost/sec. Because of this, its total communication cost/sec is higher than SMR based scheme. In case of the proposed scheme due to the incorporation of *λ*_*a*_ in SMR calculation and dynamic selection of optimal *δ*_th_, total communication cost/sec is the least among all the schemes discussed. So, compared to SMR based scheme, MEMO, M^3^, and WMM incur 575.11%, 105.59%, and 6.43% higher average total communication cost/sec, respectively. 

**Figure 7:**
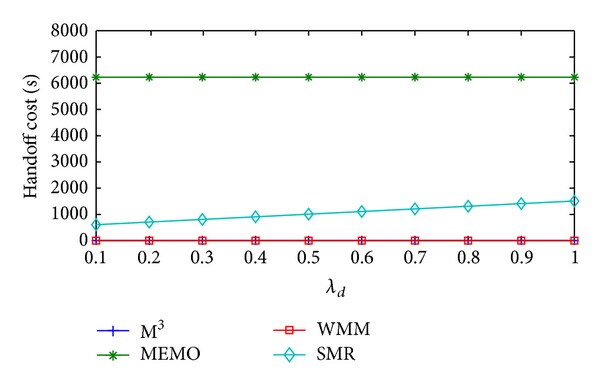
Handoff cost/sec versus *λ*_*d*_.

**Figure 8:**
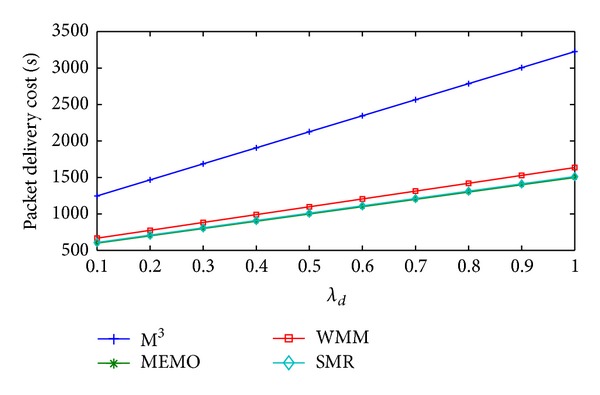
Packet delivery cost/sec versus *λ*_*d*_.

**Figure 9:**
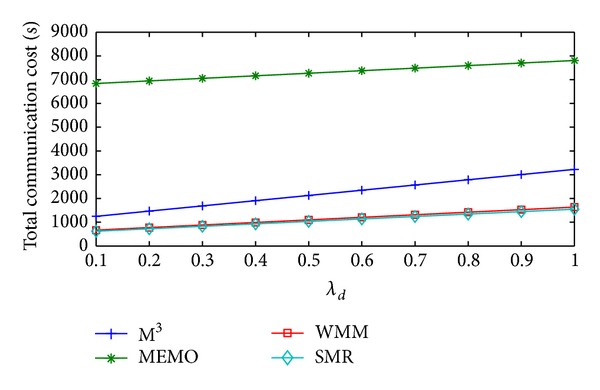
Total Communication cost/sec versus *λ*_*d*_.

Change in handoff cost/sec, packet delivery cost/sec, and total communication cost/sec of the four schemes with respect to *λ*_*d*_ are shown in Figures 7, 8, and 9, respectively. Here, the values of *λ*_*s*_ and *λ*_*a*_ are assumed to be 0.2 and 0.5, respectively. With the increase in *λ*_*d*_ handoff cost/sec and packet delivery cost/sec of all the four schemes will show the similar behavior as it was in case of *λ*_*a*_. The reasons for that are also similar. Average handoff cost/sec of MEMO is 45596.39% higher than SMR based scheme. But compared to SMR based scheme, M^3^ and WMM incur 99.70% and 99.71% less average handoff cost/sec, respectively. On the other hand, average packet delivery cost/sec of MEMO is 0.99% less than SMR based scheme. But, in comparison with SMR based scheme, M^3^ and WMM has 101.83% and 8.58% higher average packet delivery cost/sec, respectively. Other than, handoff cost/sec and packet delivery cost/sec, query cost/sec is also another factor that will affect total communication cost/sec of MEMO and SMR based scheme with the increase in *λ*_*d*_. In case of MEMO, if an MC wants to initiate a session and its host MR does not have a route to the destination, the host MR of the MC broadcasts query message to all the MRs and GW of WMN. This cost increases as *λ*_*d*_ increases and thus total communication/sec will also increase. In case of the SMR based scheme, before initiating a session the current MR of the MC sends query message to the GW and as *λ*_*d*_ increases, query cost/sec will increase. So, total communication cost/sec will increase as *λ*_*d*_ increases but it will be the least due to dynamic selection of optimal threshold SMR value. Average total communication cost/sec of MEMO, M^3^, and WMM is 574.8%, 106.04%, and 6.12% higher than SMR based scheme, respectively.

**Figure 10:**
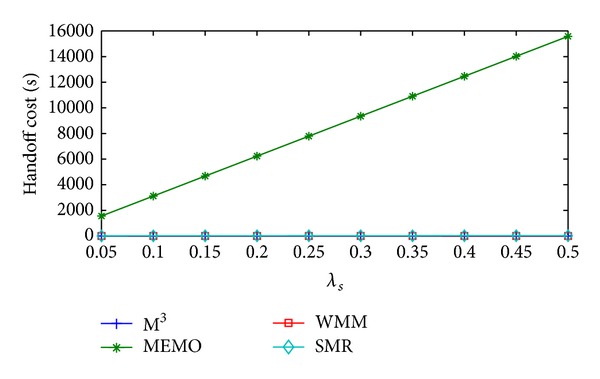
Handoff cost/sec versus *λ*_*s*_.

Figure 10 shows the change in packet delivery cost/sec of MEMO, M^3^, WMM, and SMR based scheme with respect to *λ*_*s*_. Here, both *λ*_*a*_ and *λ*_*d*_ are assumed to be 0.5. In MEMO, if *λ*_*s*_ increases, number handoff/sec will also increase and the host MR has to send and receive more route request and route reply. So, handoff cost/sec will increase at higher rate as *λ*_*s*_ increases. The handoff cost/sec of M^3^ and WMM will not increase much as *λ*_*s*_ increases. This is because in M^3^ periodic location update messages are sent and in WMM no explicit location update is sent. In case of SMR based scheme with the increase in *λ*_*s*_ there is slight increase handoff cost/sec because of dynamic selection of optimal threshold value *δ*_th_. But the rate of increase is very small. Average handoff cost/sec of MEMO is 56604.93% higher than SMR based scheme. On the other hand, compared to SMR based scheme M^3^ and WMM incur 99.63% and 99.64% less average handoff cost/sec, respectively.

**Figure 11:**
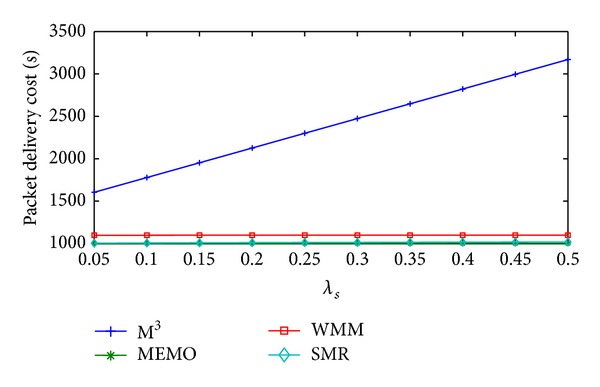
Packet delivery cost/sec versus *λ*_*s*_.

Figure 11 shows the change in packet delivery cost/sec of MEMO, M^3^, WMM, and SMR based scheme with respect to *λ*_*s*_. MEMO does not use any forward pointer, so with increase in *λ*_*s*_ the packet delivery cost/sec will be constant. In M^3^ as *λ*_*s*_ increases, the MC performs more number of handoffs between two consecutive location updates. Thus the packets have to go through longer forward chain and packet delivery cost/sec will increase. Packet delivery cost/sec of WMM increases a little as *λ*_*s*_ increases. This is because here session arrival and departure rate is assumed to be constant. It results in a constant but small time interval between two consecutive location updates and with the increase in *λ*_*s*_ the packets will traverse little larger forward chain. In SMR based scheme the value of *δ*_th_ is adjusted dynamically. Though *λ*_*s*_ increases, the forward chain does not increase much and as a result there will be very little increase in packet delivery cost/sec. Compared to the SMR based scheme, M^3^ and WMM have 135.85% and 8.47% higher average packet delivery cost/sec, respectively. But MEMO has 1.2% less average packet delivery cost/sec than SMR based scheme.

**Figure 12:**
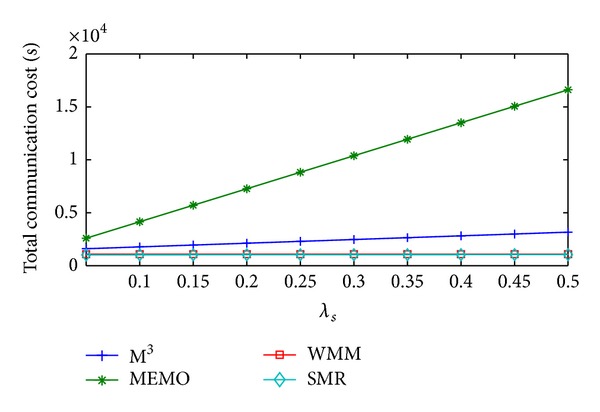
Total communication cost/sec versus *λ*_*s*_.

Figure 12 shows the change in packet total communication cost/sec of MEMO, M^3^, WMM, and SMR based scheme with respect to *λ*_*s*_. Total communication cost/sec incurred by MEMO will increase at the highest rate as *λ*_*s*_ increases. This is because of its highest increase rate of handoff cost/sec. The increase in total communication cost/sec of M^3^ is higher than WMM and SMR based scheme as *λ*_*s*_ increases. Highest increase rate of packet delivery cost/sec incurred by M^3^ is the main reason behind this. WMM's total communication cost/sec remains higher than SMR based scheme as *λ*_*s*_ increases. This is because it has higher packet delivery cost/sec and significant amount of query cost/sec. In SMR based scheme both handoff cost/sec and packet delivery cost/sec are optimal, so the total communication cost/sec of SMR remains the least as *λ*_*s*_  increases. Compared to SMR based scheme average total communication cost/sec of MEMO, M^3^, and WMM is 826.09%, 130.14%, and 5.85% higher, respectively. 

**Figure 13:**
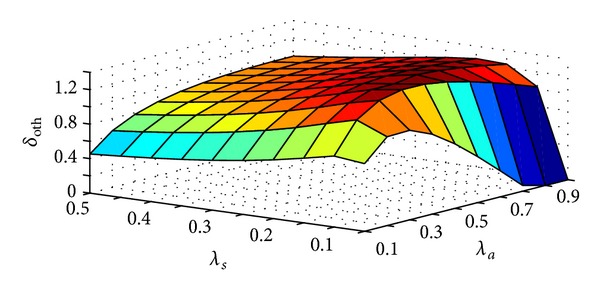
Effect of *λ*_*s*_ and *λ*_*a*_ on *δ*_oth_.

**Figure 14:**
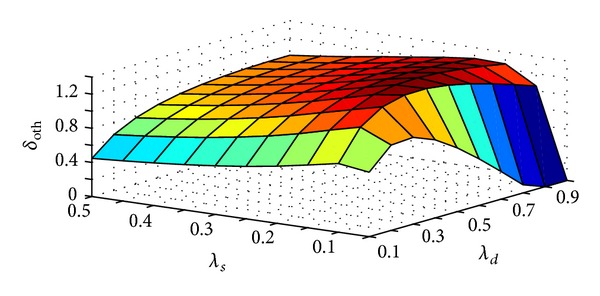
Effect of *λ*_*s*_ and *λ*_*d*_ on *δ*_oth_.

Two components of total communication cost/sec: handoff cost/sec and packet delivery cost/sec, are opposite to each other. It means, if for an MC the earlier increases the later will decrease and vice versa. Thus both of the costs need to be optimized so that total communication cost/sec is minimized. The scheme was proposed to reduce total communication cost/sec. Optimal threshold SMR (*δ*_oth_) value is determined dynamically and it is set as *δ*_th_ for minimum total communication cost/sec.  Figure 13 shows the effect of change in *λ*_*a*_ and *λ*_*s*_ on *δ*_oth_. If the value of *λ*_*a*_ increases SMR value will also increase. Initially optimal threshold SMR value (*δ*_oth_) increases with increase in *λ*_*a*_ to avoid drastic decrease in forward chain length causing very high handoff cost/sec. At higher values of *λ*_*a*_, *δ*_oth_ decreases as *λ*_*a*_ increases. It helps to keep the packet delivery cost/sec as minimum because it has become more important component of total communication cost/sec with higher *λ*_*a*_. On the other hand, if *λ*_*s*_ increases more handoffs will take place and to reduce high handoff cost/sec longer forward chain is desirable. But too long forward chain will result in higher packet delivery cost/sec. If the value of *λ*_*s*_ increases SMR value will decrease. For small value of *λ*_*s*_ optimal threshold SMR value (*δ*_oth_) increases with increase in *λ*_*s*_. This reduces frequency of location update operation. For small value of *λ*_*s*_, less frequency of location update operation will not cause drastic increase in forward chain length. So, both handoff cost/sec and packet delivery cost/sec are balanced such that total communication cost/sec is the minimum. In later section of the figure, when *λ*_*s*_ is higher, *δ*_oth_ decreases as *λ*_*s*_ increases. This is to avoid very long forward chain which in turn reduces packet delivery cost/sec. Thus minimizing total communication cost/sec.  Figure 14 shows effect of change in *λ*_*d*_ and *λ*_*s*_ on *δ*_oth_. From the figure it can be observed that the change in *δ*_oth_ due to variation in *λ*_*d*_ and *λ*_*s*_ follows the same trend as in case of Figure 13 and the reasons for that are also similar.

## 8. Conclusion and Future Work

In this paper, a SMR based mobility management scheme has been proposed. The proposed scheme is per user based. Each MC periodically calculates optimal value of threshold SMR for minimum total communication cost/sec. The value of *δ*_th_ is adjusted dynamically based on that optimal threshold SMR value. 

An analytical model for performance evaluation of the proposed scheme has been developed. The proposed scheme is also compared with MEMO, M^3^, and WMM. Analytical results show that the proposed scheme performs better than the rest of the three schemes. The variation in average forward chain length with respect to increase in session arrival rate, session departure rate, and mobility rate is also analyzed in this paper. 

Investigation on the possibility of extending the proposed scheme for WMNs with more than one GWs remains as future work. In addition exploration possibility for use of caching in the proposed scheme to reduce signaling overhead also remains as future work.

## Figures and Tables

**Table 1:** Parameters and their interpretations for mathematical modeling and analysis.

Parameter	Interpretation
*M*	Total number of MRs in the network
*α*	Average distance (hop count) from an arbitrary MR to the gateway
*β*	The average distance between two arbitrary MRs
*γ*	Per hop communication latency
*δ*_th_	Threshold value for SMR
*λ*_*p*_	Average number of packets in a session per time unit
*t*_*M*3_	Time interval between two consecutive location updates in *M* ^3^ scheme
*N*_active_	Average number of corresponding MCs in the WMN per MC
*ω*	Rate of reconnection when an MC switches from sleep mode back to active mode
*λ*_sc_	Average mobility rate of corresponding MC
*λ*_ac_	Average session arrival rate of corresponding MC
*λ*_dc_	Average session departure rate of corresponding MC
*δ*_thc_	Average threshold value for SMR of corresponding MC
*r*_inter_	Percentage of downlink packets per Internet session
*r*_intra_	Percentage of downlink packets per Intranet session
*I*_*a*_	Probability that an arriving session to an MC be an Internet session
*I*_*d*_	Probability that a departing session from an MC be an Internet session
*p*_*g*_	Average probability that current MR of MC does not know the location information of destination MC
*p*_*r*_	Average probability that an MR broadcasts a network control message in its neighborhood
*p*_*q*_	Average probability that the location query procedure is executed in WMM scheme

**Table 2:** Default parameter values.

Symbol	Value
*γ*	0.1
*λ*_*p*_	200
*α*	50
*M*	1000
*P*_*r*_	0.5
*β*	50
*P*_*q*_	0.1
*P*_*g*_	0.5
*t*_*M*3_	1200
*C*	0.4
*N*_active_	30
*I*_*a*_	0.8
*I*_*d*_	0.7
*r*_inter_	0.8
*r*_intra_	0.5
*ω*_*w*_	1/1200
*ω*_*s*_	1/600
*λ*_sc_	0.3
*λ*_ac_	0.05
*λ*_dc_	0.5
*δ*_thc_	0.36
